# GPs’ perceptions of teaching methods in shared decision-making training: a qualitative study

**DOI:** 10.3399/BJGP.2022.0194

**Published:** 2022-10-18

**Authors:** Taona Nyamapfene, Joanne Butterworth, Haider Merchant, Mike Eaton

**Affiliations:** University of Exeter Medical School, University of Exeter, Exeter; Royal Cornwall Hospital NHS Trust, Truro.; University of Exeter Medical School, University of Exeter, Exeter.; Royal Cornwall Hospital NHS Trust, Truro.; University of Exeter Medical School, University of Exeter, Exeter.

**Keywords:** continuing professional development, general practice, postgraduate education, primary health care, qualitative research, shared decision making

## Abstract

**Background:**

Although shared decision making (SDM) is key to delivering patient-centred care, there are barriers to GPs implementing SDM in practice. SDM training is undergoing development by organisations, including the Royal College of General Practitioners. However, GPs’ perceptions of the delivery of SDM training in general practice remain largely unexplored.

**Aim:**

To explore GPs’ perceptions of teaching methods in SDM training.

**Design and setting:**

Qualitative study of GPs with teaching roles at the University of Exeter Medical School.

**Method:**

Purposive sampling recruited 14 GPs. Semi-structured interviews explored their SDM educational experiences. Data were analysed using thematic framework analysis.

**Results:**

Three themes were identified. The GPs described role-play, receiving feedback, and on-the-job learning as modes of delivering SDM training that mostly informed their SDM in clinical practice positively. Learning from knowledgeable individuals and using realistic patient cases were perceived as beneficial components of SDM learning, although most learning occurred implicitly through reflections on their clinical experiences. The GPs identified that their training on SDM should reflect the uncertainty that is present when sharing decisions with patients in real-life general practice consultations. GPs also identified the targeting of individual GPs’ SDM learning needs and explanation of the potential benefits of SDM on consultation outcomes as important methods to facilitate the implementation of SDM in practice.

**Conclusion:**

To the authors’ knowledge, this is the first UK study to explore GPs’ perceptions of SDM training and provide recommendations for practice. As SDM occurs in partnership with patients, further research should obtain and incorporate patients’ views alongside those of GPs in the evaluation of future programmes.

## INTRODUCTION

Effective shared decision making (SDM), whereby clinicians work collaboratively with patients to make clinical decisions, is a key component of patient-centred care.^1^^,^^2^ SDM has been shown to increase patients’ knowledge regarding their health conditions and their satisfaction with treatments, while optimising patient outcomes such as quality of life.^3^^,^^4^

In 2021, the National Institute for Health and Care Excellence (NICE) developed guidelines to help embed SDM within clinical practice.^2^^,^^5^ Recent initiatives within the NHS have also supported the launch of organisations such as the Personalised Care Institute, which supports and provides education on SDM for clinicians.^6^ Thus, SDM is an important topic within health care in the UK.

Factors including patients’ preparedness to participate in their health care^7^ and clinicians’ reluctance to acknowledge patients’ preferences for involvement^8^ can have an impact on the implementation of SDM. Additionally, SDM may be influenced by patients’ trust in their GP, and other patient-reported factors that are associated with the development of a therapeutic patient–GP relationship.^9^ In previous research, patients have highlighted factors such as the clinician-to-patient power balance and the lack of support placed on meeting their informational needs as challenges to applying SDM in practice.^10^ In the UK, the availability of training opportunities for clinicians, incentivisation, and professional attitudes towards SDM have also been identified as challenges affecting the implementation of SDM in primary and secondary care.^11^

In the UK, SDM is assessed in the Membership of the Royal College of General Practitioners (MRCGP) examination.^12^ GPs can also access SDM training through various avenues including online learning (for example, NHS Health Education England’s SDM e-learning resource),^13^ and face-to-face courses.^14^ However, the aforementioned barriers to SDM in practice, and the lack of pragmatic guidance on SDM for clinicians’ research, suggest that current resources are not sufficient to support SDM education for GPs.^11^^,^^15^^,^^16^ Nonetheless, collaborative action between organisations, including Health Education England and the Royal College of General Practitioners, is being undertaken to improve the development of SDM training programmes.^17^ Research on SDM training for clinicians is needed to inform the development of these training programmes and improve SDM training for GPs and other clinicians.

SDM training can be delivered using many teaching methods (Box 1).^18^^–^^24^ The use of these teaching methods in SDM interventions has been explored by clinicians from a variety of medical specialties.^11^^,^^25^^–^^27^ However, evidence on these educational approaches based on GPs’ experiences in practice is lacking.^28^ For example, roleplaying and interactive learning in SDM training have previously been favoured by clinicians, but there is limited knowledge on how such teaching methods are perceived by GPs.^11^^,^^25^^,^^27^ Therefore, this qualitative study explored GPs’ perceptions about teaching methods used in SDM training and provides insight into their strengths and weaknesses when translating learning into practice, as experienced by GPs. In doing so, this study informs the implementation of SDM as a core component of person-centred, personalised care within general practice consultations.

**Box 1.** Teaching strategies used in the delivery of SDM training

**Teaching strategies used in SDM training**	**Example**
In-person didactics	Lecture providing an overview of SDM^18^
Standardised patient	Use of a standardised patient case to practise the steps in SDM^19^
Roleplay	Roleplay on using SDM in a consultation with a patient^20^
Group discussion	Discussion of patient cases that clinicians have previously found challenging^21^
Online didactics	Online tutorial with modules on SDM^22^
Feedback/debriefing	Peer-to-peer evaluation of practice consultations using SDM^23^
Provision of resource for clinical practice	Tool with open-ended questions that clinicians can use to aid SDM in consultations with patients^24^

*SDM = shared decision making.*

**Table:** How this fits in

This study identifies new strategies that can actively improve GPs’ engagement with SDM training to facilitate their implementation and maintenance of SDM in practice. Future SDM training for GPs should incorporate the challenges of real consultations, including complexity, clinical uncertainty, and time pressure. To facilitate GPs to identify limitations within their individual SDM, explicit SDM learning opportunities that are tailored towards GPs’ individual learning needs and that communicate the benefits of SDM are necessary. Incorporating the views of patients in the evaluation of future SDM training programmes would further add to the existing body of research and enhance the delivery of SDM in general practice, thereby improving person-centred care.

## METHOD

### Design

A qualitative interview study was undertaken in UK general practices using a phenomenological approach. This study is reported according to the Standards for Reporting Qualitative Research to ensure transparency.^29^

### Recruitment

Fully qualified, practising GPs with teaching roles across University of Exeter Medical School (UEMS) localities were sought. Snowball sampling, aided by UEMS faculty leads and affiliated GPs, provided a list of GPs with teaching roles at UEMS.^30^ Using purposive sampling, 69 GPs were contacted and provided with a participant information sheet and a consent form via email.

Phenomenological studies have been successfully conducted with <10 participants,^31^ and sample sizes of 12–16 individuals have been shown to provide accurate information in qualitative studies.^32^ Therefore, a pre-determined sample size of 15 was selected, with the intention to review this based on the extent to which new data contributed to generating new themes and the completeness of the themes as perceived by the authors.^33^

### Data collection

Semi-structured interviews between February 2021 and March 2021 explored each individual’s experiences of SDM training.^34^ These were conducted on Zoom by the first author, a UEMS student, following consultation with qualitative research advisers (academics who conduct qualitative research within health and social care). An interview topic guide was developed and modified after two pilot interviews to ensure interview questions focused on SDM education rather than SDM in general (Supplementary Appendix S1). During the interviews, Microsoft PowerPoint slides containing skills that encompass SDM as defined by NICE and NHS England (Supplementary Appendix S1) were used to help GPs’ recall what SDM consisted of and prior experiences of SDM training.^1^^,^^35^

The interviews averaged 35 min and were video- and audiorecorded. The interviews were then transcribed verbatim and checked for accuracy by the first author. Participant confidentiality was maintained through anonymisation of the transcripts, removal of personally identifiable information, and secure storage of the data.

### Data analysis

Thematic analysis using the framework approach enabled the data to be summarised within matrices.^36^ To allow a growing understanding of the data as the interviews progressed, data were analysed inductively and in parallel to data collection.^34^ An audit trail, including field notes, was employed to record decisions made during the study.

The first and third author independently analysed the first three transcripts using line-by-line coding to build a coding framework.^36^^,^^37^ Intercoder disagreements were resolved through discussion and revisiting the transcripts. The first author applied the coding framework to all the transcripts using the NVivo (version 12) qualitative analysis software.^38^ New codes were added as data collection continued. Themes were identified through repeated reading of the transcripts to gain an in-depth understanding of the participants’ experiences and as data collection continued. After 14 interviews, significant replication of themes was noted within data analysis, with minimal emergence of new concepts.^39^ Thus, following discussion with the co-authors, it was deemed that subsequent interviews would be of limited value to the overarching themes as it was likely that saturation had been reached. The final themes were agreed on through discussions with the co-authors.

## RESULTS

Fourteen GPs were interviewed. The participant demographics are summarised in Table 1. The findings are presented under three main themes: modes of SDM training delivery, perceptions of beneficial components of SDM learning, and how training can facilitate the implementation of SDM in practice.

**Table 1.** The participant demographics (*N* = 14)

**Characteristic**	***n***
**Age, years**	
20–29	1
30–39	3
40–49	4
50–59	2
60–69	4

**Sex**	
Female	6
Male	8

**Years as a fully qualified GP**	
1–10	5
11–20	3
21–30	2
31–40	4

**Years in higher education teaching**	
1–10	7
11–20	5
21–30	1
31–40	1

**Ratio of GP and teaching commitments**	
GP work = teaching work	4
GP work > teaching work	8
Teaching work > GP work	2

**Teaching responsibilities**	
University teaching only	7
University teaching and GP trainer	5
University teaching and research	2

**Locality**	
Exeter	10
Cornwall	2
Torbay	1
North Devon	1

**Postgraduate qualifications**	
1–3	7
4–6	5
7–9	2

**Clinical education qualification (MSc or PGCE)**	
Yes	10
No	4

### Modes of SDM training delivery

When reflecting on their general practice training, all of the participants described learning SDM through a variety of methods. They expressed strong views about learning through roleplay, feedback, didactic teaching (instructional methods of teaching), and on-the-job learning (outlined below). Other teaching methods that were discussed less frequently are summarised in Box 2 along with participants’ views on their relative strengths and limitations.

**Box 2.** GPs’ perceptions of the strengths and limitations of other, less discussed teaching methods used in SDM training

**Teaching method**	**Strengths**	**Limitations**
Group discussion	Allows communication with colleagues in confidence Enables learning from others’ approaches	• Requires a facilitator to enable useful learning
Analysis of own recorded performance in practice	Increases self-awareness Facilitates identification of areas for improvement Accounts for non-verbal communication skills, which influence overall SDM	Recorded performance may not reflect day-to-day performance Reviewing the recordings may be uncomfortable
Being observed and observing other clinicians in practice	Facilitates learning from more experienced clinicians Enables feedback on own SDM performance	May not reflect natural interactions with patients May feel patronising for senior GPs
Small group learning	Creates a supportive learning environment	Requires a good facilitator to establish an effective group dynamic
Online learning	Online tutorials may be useful when face-to-face sessions are not possible Can be used to communicate SDM theory Could lead to passive learning and poor knowledge retention	Analysis of SDM performances may be limited in comparison with face-to-face training

*SDM = shared decision making.*

Although regarded by some participants as intimidating, many participants valued roleplay with peers or actors in enabling them to trial and assess the impact of different approaches of SDM in a safe environment. With actors, they valued dissecting their performance after the roleplay and receiving feedback from the actor on what had gone well, what had not gone well, and how performance made the actor, in their role as patient, feel. However, although most participants felt that roleplay with their peers helped them in developing their style of communication, they found feedback from this to be lacking in honest criticism compared with that from actors. Receiving feedback on their SDM performance in roleplays, recorded consultations, and observed consultations provided the participants with an insight into their own SDM performance. They valued objective feedback from real consultations, which they perceived to be more representative of their true performance in practice; and from sources whom they deemed credible, such as more experienced GPs:

*‘Erm,* [with roleplay] *you can change things. So, you can watch someone doing something, and the people observing can give feedback on what they did and didn’t do so well. And then you can rerun the consultation.’*

(GP3, male, aged 49 years)

*‘Because it was a live, real experience and you were really doing it* [SDM], *then that’s* [feedback] *quite powerful. Um* [pause] *, you sort of feel like you’re learning, not necessarily from an expert, but from somebody who’s done this for a lot longer than you have, and who’s probably better at it.’*

(GP11, female, aged 41 years)

A few participants found didactic teaching, in the form of consultation models, research evidence, and psychodynamic theories, to be important tools in understanding SDM, with some participants describing these as tools they would refer to in challenging consultations. However, the participants mostly associated didactic teaching with poor knowledge retention and engagement. Thus, they suggested combining didactic teaching with active teaching methods such as practice and group discussion:

*‘It’s in those really difficult consultations* [pause] *if you have a theory to fall back on, rather than just your personal experience, that could help you get to a better endpoint.’*

(GP5, male, aged 35 years)

*‘It’s all very well, reading something, but I think you have to make a conscious decision to actually turn that into activity, or it* [learning] *doesn’t happen.’*

(GP8, female, aged 49 years)

However, many participants described on-the-job learning as the mainstay method of learning SDM, particularly after completing GP training:

*‘But actually, in reality, probably the most learning is stuff that I would add myself through my own reflection of patient interactions* […] *So actually, most of the embedded changes in practice came out of my own experience, and the adjustments and changes that I would have put in place myself.’*

(GP4, male, aged 59 years)

Therefore, although the participants acknowledged teaching methods such as roleplay, feedback, and didactic teaching in their experiences of SDM training, they reported that most of their SDM learning had subsequently occurred on the job, through reflective practice.

### Perceptions of beneficial components of SDM learning

The participants viewed learning on SDM to be most beneficial when it reflected their encounters with patients in clinical practice and included knowledgeable others. They also felt that learning about SDM needed to be more explicit within their continuing professional development (CPD).

The participants reported that they were more likely to perceive new SDM skills as relevant to their CPD when the learning they received incorporated real patient cases that they could identify with from their own practice:

*‘I think there’s a sort of tendency, particularly for a lot of GPs who are very busy. They just trundle on as they are, with their own style, and it takes something to stop them and make them reflect. Erm, and so something that’s relevant to them or reminds them of a patient that they’ve seen, or an outcome that they have seen, is much more likely to have an impact on them.’*

(GP10, female, aged 34 years)

Many participants had appreciated previous relationships with their GP trainers and discussed ongoing relationships with their peers, with whom they reviewed challenging consultations. These relationships enabled learning through mentoring and guided evaluation of their performance:

*‘She* [GP trainer] *talked to me about everything* [emphasis]. *She watched me, I videoed myself, we went through the videos, and it would be all about how I took a history and then how I then came up with a management plan and how to share it with patients* […] *She was very, very involved in that and we would break it down and come up with phrases together.’*

(GP7, female, aged 43 years)

*‘I think currently and probably for the last number of years since completing training, it’s* [small group with colleagues] *probably the most influential method of affecting practice. Erm, not only in terms of just support as a practitioner, but also just in being able to share difficulties, sharing management queries, erm picking up different resources that different people are using.’*

(GP8, female, aged 49 years)

As most participants agreed that after completing GP training SDM was predominantly learned implicitly through reflection on clinical experiences, they suggested that SDM training for qualified GPs should be formalised. Nonetheless, these participants expressed an understanding that factors such as time, financial, and clinical constraints act as barriers to engaging qualified GPs in formal SDM teaching:

*‘It was happening without you realising, as part of the consultation. You were learning about it without realising* […] *I think it would be better to formalise it. So* [that] *you actually can be more mindful* [that] *you’re doing it.’*

(GP15, male, aged 37 years)

*‘So many things in GP* [general practice] *come down to two things* [pause] *protected time and money. If you don’t have the protected time, it doesn’t get done. The clinical pressure and load to see patients is so great* […] *So you need processes that are absolutely cast in stone.’*

(GP13, male, aged 53 years)

Thus, the participants valued learning SDM through content that was relevant to their encounters in practice and from individuals such as their colleagues but shared a need for explicit SDM training post-qualification.

### How training can facilitate the implementation of SDM in practice

When discussing the translation of learning about SDM into SDM practice, the participants identified features that they perceived to be important in facilitating and maintaining SDM in practice. They felt that, to encourage GPs’ participation and acquisition of new skills, SDM training should convey the potential benefits of successful SDM and be tailored to individual GPs’ learning needs to encourage participation. There was also a general consensus that training should prepare GPs for implementing SDM within the constraints of real consultations.

Several participants believed that training would be more likely to be adopted and achieve a lasting impact on GPs’ practice if it communicated evidence on the positive outcomes of SDM:

*‘I think what would help a lot of people is to understand the reasoning behind it* [SDM] *. So, for example, we know that if you can involve patients in their decision making, that they are more likely to comply with treatment, they are more likely to come back and review with you, and their outcomes are more likely to be better. And I think if, for a lot of people, if you could emphasise the reason why shared decision making is good, people will be more naturally inclined to do it.’*

(GP10, female, aged 34 years)

To additionally improve their SDM in consultations, the participants valued training that revealed deficiencies within their personal SDM practice and addressed these as individualised learning needs. Such training enabled them to recognise unique areas to improve on:

*‘My consultation skills were improved, and my failings were brought to me. Which was important. That’s the way you learn. When I hadn’t got the right body language or I hadn’t shared decisions then* [pause] *I may not have noticed that, but then my observer would bring that up*.’

(GP7, female, aged 43 years)

Notably, most participants reported a mismatch between their SDM performance in practice, where they felt they might underperform, when compared with training, where they felt the pressure to act as the ‘perfect doctor’. Therefore, they expressed a desire for high-fidelity training that acknowledges the challenges of implementing SDM in practice where, for example, clinical uncertainty is common and SDM *‘is not so clear cut’* (GP10, female, aged 34 years):

*‘And, over time* [with training] *, I think it’s almost like you reach a point where you become absolutely brilliant, on paper, at your communication skills. And that’s probably around the time that you sit the CSA* [Clinical Skills Assessment] *. And then after that, you realise that you cannot achieve that level of exceptional communication skills in the real world, and you have to merge it with practically what gets you through a busy surgery.’*

(GP10, female, aged 34 years)

*‘I think that the important thing is that it* [SDM training] *is as authentic as possible. Sort of, it matches real-life experience as much as possible. It allows for er the greyness. Because things don’t fall into er particular patterns. There would be sort of areas that go well, areas go less well. You’re not going to have the ideal consultation.’*

(GP4, male, aged 59 years)

## DISCUSSION

### Summary

The participants expressed that, once they were practising as qualified GPs, SDM was largely learned implicitly from their experiences within general practice. However, reflecting on their knowledge of GP training programmes, they valued roleplay, feedback, and on-the-job learning to inform changes in their SDM performance. Despite being unpopular, didactic teaching in the form of psychodynamic theories and research evidence are useful in scaffolding an understanding of SDM and providing a framework for the participants to refer to in challenging consultations. Some participants suggested that SDM learning for qualified GPs should be made more explicit through formalised teaching, but financial, time, and clinical pressures were noted as potential barriers.

The participants foresaw the potential benefits of SDM training based on real general practice and peer feedback for qualified GPs. To promote their engagement with training and application of learning, they recommended that training should communicate the benefits of SDM in practice and be tailorable to the learning needs of individual GPs. Finally, to prepare GPs to implement SDM, they expressed that interventions to train GPs in SDM should reflect the complexity of general practice consultations.

### Strengths and limitations

To the authors’ knowledge, this is the first UK study to explore GPs’ perceptions on the implementation of SDM training in UK general practice. This study highlights the attributes of SDM training that promote the adoption, implementation, and maintenance of these skills, helping to shape future SDM training programmes for GPs.

Purposive sampling enabled the sample to be varied by age, sex, and experience in general practice and teaching, resulting in a wide range of views.^31^ However, the study may have attracted individuals with an interest in SDM. This may have limited the breadth of views established on this topic. Additionally, the generalisability of the study was limited by the inclusion of participants who only held teaching roles at UEMS. As such, the participants’ teaching experiences may have influenced their perceptions on the delivery of SDM education and may not fully encompass the opinions of GPs who are not engaged in higher education teaching.^40^ Nonetheless, by uniquely sampling GPs with expertise in teaching approaches, this study provides insights on SDM training from GPs who are knowledgeable on both general practice and educational methods. By exploring the participants’ experiences across several general practices and locations, the findings are transferable to the SDM experiences of GPs in the regional context, and perhaps the wider population.

Additionally, as the interviews explored the participants’ SDM experiences within and beyond their year of GP qualification (range 1986–2019), the findings draw from the variety of teaching methods experienced by the participants over this period of time. This ensured a comprehensive insight into the different methods of teaching used in SDM training.

The use of individual interviews allowed the discourse of the interviews to be tailored to the participants’ individual experiences, unlike focus groups.^41^ Conducting the interviews on Zoom enabled them to occur at participants’ convenience and the use of video allowed for non-verbal communication such as attentive listening. This enabled the interviewer to establish rapport, unlike telephone interviews in which forming rapport is more difficult.^42^^,^^43^

The potential of the participants holding different definitions for the term ‘shared decision making’, and the impact of this on the experiences that they shared was acknowledged. Consequently, the interviews began by exploring the meaning of this term to each participant and establishing a consistent definition by all participants.

Thematic framework analysis facilitated rich descriptions of each individual’s experiences, which assisted in collectively interpreting the data and considering data saturation, which was felt to have been achieved.^36^ Analysing the initial transcripts in duplicate and addressing disagreements ensured reliability and coding accuracy. Maintaining an audit trail further added to the study’s reliability and dependability.^44^

As the primary researcher is a medical student with no prior experience of GP SDM training, the impact of their background on the interpretation of the data was addressed through the use of a reflexive research diary to consider alternative perspectives and record decisions made during the study.^45^

### Comparison with existing literature

A systematic review of international SDM educational programmes for medical trainees identified that most SDM programmes combine didactic and practical teaching methods.^25^ During the ‘Making good decisions in collaboration’ (MAGIC) workshops, aimed at UK clinicians, practical teaching methods were viewed as superior to didactic teaching.^11^ The literature, therefore, concurs with the present findings and indicates a role for the use of both teaching methods together in SDM training. However, both of these studies had a broad demographic of clinicians from many specialties and so lacked the focus on GPs presented here.

A qualitative study of medical students, clinicians, and patients’ perceptions of SDM learning in clinical practice found that SDM learning occurs through implicit and informal processes.^46^ Additionally, learning SDM from other professionals such as role models was identified as valuable in learning SDM by reflective practice.^46^ However, an analysis of the MAGIC programme suggested that clinicians’ attitudes towards SDM, including the belief that they already engage in SDM in their existing practice, are a barrier to their implementation of SDM.^11^ GPs’ incorrect judgement of their SDM competence and understanding of good SDM in comparison with their current practice may limit their implicit learning and identification of true role models when learning on the job. The current study adds that training on SDM should identify and address GPs’ individual deficiencies within their SDM practice. This can facilitate GPs to recognise areas within their practice that require improvement. This can be through the provision of feedback to the GPs on their SDM from selected GP colleagues during formal learning opportunities such as observations in clinics, and group discussion to explicitly embed SDM within on-the-job learning in general practice.

An article providing guidance on incorporating SDM in routine practice suggested that increasing clinicians’ understanding of the rationale that underpins SDM can improve their attitudes towards SDM.^47^ The participants in the present study suggest communicating research evidence on the benefits of SDM through didactic teaching as a possible way to convey the rationale for SDM. Furthermore, a report on the MAGIC programme captured GPs’ and other clinicians’ views that SDM training needs to be incentivised and focused on clinicians’ needs.^16^ This is consistent with the findings of a King’s Fund report on promoting SDM in the NHS that recommended that incentives are required to motivate clinicians to implement SDM.^48^ The reports fail to stipulate incentives that target the uptake of SDM practices in GPs specifically. However, the participants in this study also identify communicating the potential positive impact of SDM to GPs as a possible incentive to encourage GPs’ implementation of SDM.

This study adds to the existing literature by uniquely identifying that didactic teaching on SDM should communicate the rationale for SDM, while the practical components should be based on real patient scenarios that illustrate the uncertainty of general practice consultations. Previous research has identified limited time within clinical encounters as a barrier to dialogues between clinicians and patients that facilitate SDM.^49^ The present study emphasises that the practical component of SDM training could be enhanced by including scenarios that address real-life challenges including time constraints and clinical uncertainty. Consequently, by reflecting the complexity of general practice consultations, this approach might help mitigate barriers to SDM in general practice consultations.

### Implications for research and practice

This study has explored SDM training for GPs through the perspectives of GPs, a key stakeholder group. Health organisations and policymakers must ensure that SDM training for GPs enables the practical application of SDM that reflects the uncertainty and challenges of general practice consultations. Training should also address GPs’ individual learning needs. To promote the long-term application of SDM in practice, training messages should be reinforced by conveying research evidence on the benefits of SDM on consultation outcomes.

The present findings inform GPs to actively seek out and apply favourable modes of delivery and components of SDM training when pursuing their individual CPD. Future research should establish barriers and enablers to SDM education and modalities of SDM training that are favoured by learners within the undergraduate and postgraduate medical curricula. This can help determine how SDM can be best incorporated within these curricula.

This study also provides patients with evidence of the medical research community’s commitment to person-centred care within general practice, with a view to improving patient experiences of care.^50^ To fully inform the development of future SDM training programmes, future research should include patients’ perspectives alongside those of GPs in the evaluation of their delivery and implementation. As SDM occurs in partnership with patients, ascertaining patients’ views is paramount to improving patient experiences of care and health outcomes.^50^ Incorporating both perspectives will help create a curriculum for SDM for qualified GPs, to facilitate the effective delivery of SDM as a core component of person-centred care.^1^

## References

[1] National Institute for Health and Care Excellence (2019). Shared decision making KTT23.

[2] Elwyn G, Edwards A, Gwyn R, Grol R (1999). Towards a feasible model for shared decision making: focus group study with general practice registrars. BMJ.

[3] Amir N, McCarthy HJ, Tong A (2021). A working partnership: a review of shared decision-making in nephrology. Nephrology (Carlton).

[4] Coronado-Vazquez V, Canet-Fajas C, Delgado-Marroquin MT (2020). Interventions to facilitate shared decision-making using decision aids with patients in primary health care: a systematic review. Medicine (Baltimore).

[5] National Institute for Health and Care Excellence (2021). Shared decision making NG197.

[6] Royal College of General Practitioners (2020). New personalised care training hub will set standards for evidence-based education and aims to reach 75,000 health and care workers by 2024.

[7] Keij SM, van Duijn-Bakker N, Stiggelbout AM, Pieterse AH (2021). What makes a patient ready for shared decision making? A qualitative study. Patient Educ Couns.

[8] Jesse J, Shannon M, Carissa B (2019). Shared decision-making about cardiovascular disease medication in older people: a qualitative study of patient experiences in general practice. BMJ Open.

[9] Butterworth JE, Campbell JL (2014). Older patients and their GPs: shared decision making in enhancing trust. Br J Gen Pract.

[10] Joseph-Williams N, Elwyn G, Edwards A (2014). Knowledge is not power for patients: a systematic review and thematic synthesis of patient-reported barriers and facilitators to shared decision making. Patient Educ Couns.

[11] Joseph-Williams N, Lloyd A, Edwards A (2017). Implementing shared decision making in the NHS: lessons from the MAGIC programme. BMJ.

[12] Royal College of General Practitioners MRCGP: Recorded Consultation Assessment (RCA). https://www.rcgp.org.uk/mrcgp-exams/recorded-consultation-assessment.

[13] NHS Health Education England Shared decision making. https://www.e-lfh.org.uk/programmes/shared-decision-making.

[14] CPD Certification Service Mastering shared decision making. https://www.cpduk.co.uk/courses/medical-protection-society-mastering-shared-decision-making.

[15] Staveley I, Sullivan P (2015). We need more guidance on shared decision making. Br J Gen Pract.

[16] Health Foundation (2013). Implementing shared decision making: clinical teams’ experiences of implementing shared decision making as part of the MAGIC programme.

[17] National Institute for Health and Care Excellence Shared decision making collaborative — an action plan.

[18] Bhatt NR, Doherty EM, Mansour E (2016). Impact of a clinical decision making module on the attitudes and perceptions of surgical trainees. ANZ J Surg.

[19] Rusiecki J, Schell J, Rothenberger S (2018). An innovative shared decision-making curriculum for internal medicine residents: findings from the University of Pittsburgh medical center. Acad Med.

[20] Sanders ARJ, Bensing JM, Essed M (2017). Does training general practitioners result in more shared decision making during consultations?. Patient Educ Couns.

[21] Wollny A, Altiner A, Daubmann A (2019). Patient-centered communication and shared decision making to reduce HbA1c levels of patients with poorly controlled type 2 diabetes mellitus — results of the cluster-randomized controlled DEBATE trial. BMC Fam Pract.

[22] Légaré F, Labrecque M, Cauchon M (2012). Training family physicians in shared decision-making to reduce the overuse of antibiotics in acute respiratory infections: a cluster randomized trial. CMAJ.

[23] Tilburgs B, Koopmans R, Vernooij-Dassen M (2020). Educating Dutch general practitioners in dementia advance care planning: a cluster randomized controlled trial. J Am Med Dir Assoc.

[24] Sanders ARJ, Bensing JM, Magnee T (2018). The effectiveness of shared decision-making followed by positive reinforcement on physical disability in the long-term follow-up of patients with nonspecific low back pain in primary care: a clustered randomised controlled trial. BMC Fam Pract.

[25] Singh Ospina N, Toloza FJK, Barrera F (2020). Educational programs to teach shared decision making to medical trainees: a systematic review. Patient Educ Couns.

[26] Hoffmann TC, Del Mar C, Santhirapala R, Freeman A (2020). Teaching clinicians shared decision making and risk communication online: an evaluation study. BMJ Evid Based Med.

[27] Légaré F, Moumjid-Ferdjaoui N, Drolet R (2013). Core competencies for shared decision making training programs: insights from an international, interdisciplinary working group. J Contin Educ Health Prof.

[28] Oerlemans AJM, Knippenberg ML, Olthuis GJ (2021). Learning shared decision-making in clinical practice. Patient Educ Couns.

[29] O’Brien BC, Harris IB, Beckman TJ (2014). Standards for reporting qualitative research: a synthesis of recommendations. Acad Med.

[30] Naderifar M, Goli H, Ghaljaei F (2017). Snowball sampling: a purposeful method of sampling in qualitative research. Stride Dev Med Educ.

[31] Moser A, Korstjens I (2018). Series: Practical guidance to qualitative research. Part 3: sampling, data collection and analysis. Eur J Gen Pract.

[32] Romney AK, Weller SC, Batchelder WH (1986). Culture as consensus: a theory of culture and informant accuracy. Am Anthropol.

[33] Saunders B, Sim J, Kingstone T (2018). Saturation in qualitative research: exploring its conceptualization and operationalization. Qual Quant.

[34] DiCicco-Bloom B, Crabtree BF (2006). The qualitative research interview. Med Educ.

[35] NHS England, NHS Improvement (2019). Shared decision making: summary guide. https://www.england.nhs.uk/wp-content/uploads/2019/01/shared-decision-making-summary-guide-v1.pdf.

[36] Gale NK, Heath G, Cameron E (2013). Using the framework method for the analysis of qualitative data in multi-disciplinary health research. BMC Med Res Methodol.

[37] Sutton J, Austin Z (2015). Qualitative research: data collection, analysis, and management. Can J Hosp Pharm.

[38] Bazeley P, Jackson K (2013). Qualitative data analysis with NVivo.

[39] Vasileiou K, Barnett J, Thorpe S, Young T (2018). Characterising and justifying sample size sufficiency in interview-based studies: systematic analysis of qualitative health research over a 15-year period. BMC Med Res Methodol.

[40] Lewis AP, Bolden KJ (1989). General practitioners and their learning styles. J R Coll Gen Pract.

[41] Tai J, Ajjawi R (2016). Undertaking and reporting qualitative research. Clin Teach.

[42] Saarijärvi M, Bratt E-L (2021). When face-to-face interviews are not possible: tips and tricks for video, telephone, online chat, and email interviews in qualitative research. Eur J Cardiovasc Nurs.

[43] Kvale S (2007). Doing interviews.

[44] Korstjens I, Moser A (2018). Series: Practical guidance to qualitative research. Part 4: Trustworthiness and publishing. Eur J Gen Pract.

[45] Barrett A, Kajamaa A, Johnston J (2020). How to … be reflexive when conducting qualitative research. Clin Teach.

[46] Oerlemans AJM, Knippenberg ML, Olthuis GJ (2021). Learning shared decision-making in clinical practice. Patient Educ Couns.

[47] Elwyn G, Frosch D, Thomson R (2012). Shared decision making: a model for clinical practice. J Gen Intern Med.

[48] Coulter A, Collins A (2011). Making shared decision-making a reality: no decision about me, without me.

[49] Ives J, Papanikitas A, Myres P, Gregory S (2018). Shared decision making: a need for honesty?. Br J Gen Pract.

[50] Hughes TM, Merath K, Chen Q (2018). Association of shared decision-making on patient-reported health outcomes and healthcare utilization. Am J Surg.

